# The second Southern African Bird Atlas Project: Causes and consequences of geographical sampling bias

**DOI:** 10.1002/ece3.3228

**Published:** 2017-07-27

**Authors:** Sanet Hugo, Res Altwegg

**Affiliations:** ^1^ South African Institute for Aquatic Biodiversity Grahamstown South Africa; ^2^ Centre for Statistics in Ecology, Environment and Conservation Department of Statistical Sciences University of Cape Town Rondebosch South Africa; ^3^ African Climate and Development Initiative University of Cape Town Rondebosch South Africa

**Keywords:** citizen science, occupancy modeling, sampling effort, spatial bias, species accumulation curves, species distribution atlas, species distribution modeling

## Abstract

Using the Southern African Bird Atlas Project (SABAP2) as a case study, we examine the possible determinants of spatial bias in volunteer sampling effort and how well such biased data represent environmental gradients across the area covered by the atlas. For each province in South Africa, we used generalized linear mixed models to determine the combination of variables that explain spatial variation in sampling effort (number of visits per 5′ × 5′ grid cell, or “pentad”). The explanatory variables were distance to major road and exceptional birding locations or “sampling hubs,” percentage cover of protected, urban, and cultivated area, and the climate variables mean annual precipitation, winter temperatures, and summer temperatures. Further, we used the climate variables and plant biomes to define subsets of pentads representing environmental zones across South Africa, Lesotho, and Swaziland. For each environmental zone, we quantified sampling intensity, and we assessed sampling completeness with species accumulation curves fitted to the asymptotic Lomolino model. Sampling effort was highest close to sampling hubs, major roads, urban areas, and protected areas. Cultivated area and the climate variables were less important. Further, environmental zones were not evenly represented by current data and the zones varied in the amount of sampling required representing the species that are present. SABAP2 volunteers' preferences in birding locations cause spatial bias in the dataset that should be taken into account when analyzing these data. Large parts of South Africa remain underrepresented, which may restrict the kind of ecological questions that may be addressed. However, sampling bias may be improved by directing volunteers toward undersampled regions while taking into account volunteer preferences.

## 1 INTRODUCTION

Progress in macroecology, biogeography, and large‐scale conservation planning is enabled by a growing number of nonsystematically collected species distribution databases in the form of museum‐curated collections (specimen collections) and large‐scale species atlases (Robertson, Cumming, & Erasmus, 2010). Such databases, representing multiple taxa and large regional to subcontinental spatial scales, are increasing in scope (i.e., taxonomic and geographical) and detail (i.e., spatiotemporal resolution and types of information recorded). This development is aided by constant improvements in digital database management, accessibility (e.g., open source and Internet‐based data), and analysis (computing power and statistical techniques) (Boakes et al., 2010; Kelling et al., 2013). However, the adequate sampling of huge amounts of georeferenced species distribution data is a persistent challenge.

Specimen collections depend largely on professional scientists such as taxonomists, whereas species atlases, especially of conspicuous or charismatic taxa (e.g., birds or butterflies), are often organized as citizen science projects supported by hundreds of volunteer observers (Bird et al., 2014; Botts, Erasmus, & Alexander, 2011; Robertson et al., 2010; Tulloch & Szabo, 2012). Both specimen collections and species atlases tend to be inherently biased in terms of when and where contributors decide to sample (spatiotemporal bias) and the skill of contributors as data collectors (e.g., variation in identification and record keeping) (Bird et al., 2014; Boakes et al., 2010; Peterson, Navarro‐Sigüenza, & Benítez‐Díaz, 1998; Reddy & Dávalos, 2003; Robertson et al., 2010; Sastre & Lobo, 2009; Tulloch & Szabo, 2012). Several recent studies on spatial or geographical sampling bias show that sampling sites tend to be chosen based on accessibility, that is, traveling distance and ease of traveling (e.g., roads and terrain) to or within the sampling site, and on the attractiveness of a site for sampling, for example, the expectation of high biodiversity or of observing rare or charismatic species (Botts et al., 2011; Reddy & Dávalos, 2003; Romo, García‐Barros, & Lobo, 2006; Tulloch, Mustin, Possingham, Szabo, & Wilson, 2013). Citizen volunteers may also be motivated by a esthetic (e.g., scenic landscape features) and recreational factors (Tulloch et al., 2013). Consequently, a large proportion of samples originate from a small proportion of geographical space in and around residential and protected areas, whereas locations that are remote or believed to be low in biodiversity tend to be poorly sampled (Botts et al., 2011; Peterson et al., 1998; Sastre & Lobo, 2009).

If ignored, spatial sampling bias may result in distorted views of biodiversity, biogeography, and species distributions, with observed patterns of variation reflecting sampling effort rather than environmental or demographic causes (Bird et al., 2014; Botts et al., 2011; Evans, Greenwood, & Gaston, 2007). Species distribution databases are more useful if data are compiled with a standardized sampling protocol and include information about the observation process, for example, a measure of sampling effort for each record within the database (Bird et al., 2014; Guillera‐Arroita, 2017; Robertson et al., 2010). Further, species distribution databases may be designed with a variety of objectives, for example, whether sampling would attempt a wide coverage or whether sampling would be focused or stratified according to habitat or protected areas (Tulloch et al., 2013). Clear understanding of spatial sampling bias, survey objectives, and data types is essential, especially when considering that various species distribution databases, each with particular sampling methods and biases, are integrated and studied at a global scale (www.gbif.org; www.mol.org; Jetz, McPherson, & Guralnick, 2012).

Species distribution (Guisan & Zimmermann, 2000) or occupancy (Mackenzie et al., 2006) modeling techniques relate species distribution data to environmental covariates (e.g., spatial variation in climate and habitat type) to infer species spatial distributions. These techniques can account for variation in sampling effort, interpolate geographical “gaps” in the data, or predict the geographical locations that should be prioritized for additional sampling (Bird et al., 2014; Bled, Nichols, & Altwegg, 2013; Hernandez, Graham, Master, & Albert, 2006; Kramer‐Schadt et al., 2013; Phillips et al., 2009). However, these techniques are most reliable if based on repeated visits of sampling sites that represent the full range of variation in the environment (Araújo & Guisan, 2006; Bled et al., 2013; Hernandez et al., 2006; Phillips et al., 2009). Occupancy techniques, in particular, require multiple repeated visits to model the probability of detecting species that are present (Altwegg, Wheeler, & Erni, 2008; Bled et al., 2013; Broms, Hooten, Johnson, Altwegg, & Conquest, 2016; Guillera‐Arroita, 2017). Species detectability may vary due to several mechanisms, such as species traits, observer skill, survey methods and conditions, and habitat characteristics (Guillera‐Arroita, 2017). Species distribution and occupancy techniques are an actively developing field of research, and are widely and increasingly used to study species spatial distributions and range dynamics (Guillera‐Arroita, 2017; Guillera‐Arroita et al., 2015). These techniques benefit most from an environmentally stratified sampling design, rather than attempting to close geographical gaps by sampling as much area as possible but with low effort per unit area (Araújo & Guisan, 2006; Guillera‐Arroita, 2017; Kramer‐Schadt et al., 2013; Tulloch et al., 2013).

In South Africa, large‐scale species distribution databases facilitated a wealth of ecological research and conservation planning analyses (e.g., Harrison, Underhill, & Barnard, 2008), with historical and current databases including birds, frogs, mammals, butterflies, spiders, proteas, and invasive alien plants (find the host organizations at adu.org.za, www.proteaatlas.org.za and www.sanbi.org). The second Southern African Bird Atlas Project (SABAP2), which was launched in the year 2007, is arguably the most ambitious atlas project for the region in terms of scope, resolution and data volume. Citizen scientists record bird species presence at a relatively fine resolution (grid cells of 5 min latitude by 5 min longitude, termed “pentads”) within eight sub‐Saharan African countries, namely South Africa, Lesotho, Swaziland, Namibia, Botswana, Zimbabwe, Mozambique, and Kenya. By the end of May 2017, nearly 2,300 observers had conducted nearly 187,000 separate surveys, contributing more than 9.6 million records, and covering more than 17,700 pentads, and rate of contributions remain high (http://sabap2.adu.org.za/). However, current SABAP2 data show obvious and substantial spatial bias in sampling effort. Repeatedly sampled pentads comprise a small proportion of the total area and tend to be spatially clustered, forming a few well‐sampled geographical regions. Conversely, outside these well‐sampled regions, there still remain large poorly sampled geographical areas.

The second Southern African Bird Atlas Project is designed to run indefinitely with the aim of creating a valuable long‐term dataset for southern Africa. Thus, an assessment of sampling bias will provide much‐needed information for data users and future sampling endeavors, and ensure that volunteers' time and effort and their contributed data are used to full potential. Wright, Underhill, Keenec, and Knight (2015) previously studied the motivation of SABAP2 volunteers and the benefits they gain. However, a spatially explicit study of the possible causes and consequences of spatial sampling bias has not been conducted for SABAP2. Moreover, accounting for and improving observation bias contributes to developing species distribution data that are useful in global ecological studies. Similar evaluations of sampling bias could benefit other new or existing species atlases for many taxa around the world. Our aims are (1) to reveal spatially explicit determinants of variation in sampling effort in SABAP2 and (2) to illustrate variation in data representativeness among a variety of environments.

## 2 METHODS

### 2.1 Atlas characteristics

We focused on South Africa, Lesotho, and Swaziland where data are accumulating most rapidly and widely, and for which comprehensive environmental and human‐related GIS (geographical information system) datasets are available. The sampling protocol of SABAP2 was designed to standardize sampling by requesting that the volunteers record all the birds they encounter within a pentad for at least 2 hr (intensive sampling period), but no longer than five consecutive days, and that they attempt to cover all habitat types within the pentad. Volunteers are coordinated through regional atlas committees and the SABAP2 Web site (http://sabap2.adu.org.za/), which includes training materials (e.g., how to use GIS programmes and recognize pentad boundaries), workshops (e.g., bird identification), and birding events. The online submission process links records automatically to a coverage map and flags unusual (e.g., out of range) records that are then vetted by regional atlas committees. The SABAP2 database includes information on sampling effort for each pentad in terms of number of contributed species lists (i.e., one list per visit) and number of records (i.e., species sightings), as well as number of hours and days spent sampling per pentad.

Atlas data used in this study were contributed between June 2007 and the end of August 2016. For this period, about 75% of the pentads covering South Africa, Lesotho, and Swaziland were visited at least once (i.e., one or more lists contributed); however, <16% of pentads were sampled 10 times or more; that is, enough repeated visits to ensure that common species were detected with high probability, even with relatively low detectability (Guillera‐Arroita, Ridout, & Morgan, 2010). Spatial bias could be partly attributed to coordination efforts of the regional atlas committees for each province and the “birding challenges” that aim to intensively sample regions of special concern for bird biodiversity. Areas covered by birding challenges include Kruger National Park, Western Cape Province, and the four degrees latitude and longitude encompassing Gauteng and parts of the surrounding provinces (the Gauteng 4D birding challenge).

### 2.2 Determinants of spatial variation in sampling effort

**Figure 1:**
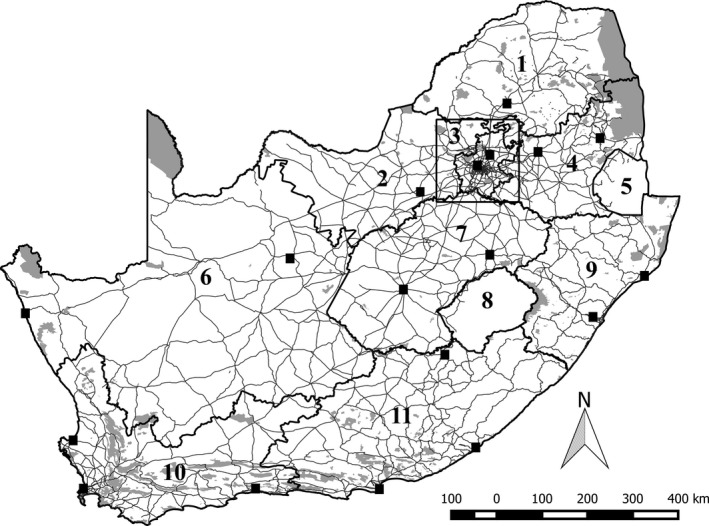
Study area: (1) Limpopo Province, (2) North West Province, (3) the four degree square comprising the Gauteng 4D birding challenge, (4) Mpumalanga Province, (5) Swaziland, (6) Northern Cape Province, (7) Free State Province, (8) Lesotho, (9) KwaZulu‐Natal Province, (10) Western Cape Province, (11) Eastern Cape Province. Thick lines indicate province and country boundaries, and fine lines indicate major roads. Black squares indicate the location of sampling hubs, that is, locations with exceptionally high sampling effort. Gray shading indicates all terrestrial formal and informal protected area (SANBI, 2010, 2011)

For this analysis, we investigated each South African province separately (Lesotho and Swaziland were not included) to account for the possible influence of the regional atlas committees and birding challenges, and to account for regional differences in level of human population density and development (Figure 1, Table S1). We investigated the entire four degrees comprising the Gauteng 4D challenge separately from the rest of the surrounding provinces to account for the increased sampling in this region (Figure 1, Table S1). We explored factors representing the accessibility (1–2) and attractiveness (3–5) of each pentad that may explain spatial variation in sampling effort.

1. The pentads with the greatest number of contributed lists are located some distance apart and correspond to the locations of major cities such as Johannesburg or small towns at popular ecotourism or birding destinations such as Lady Grey. Sampling effort tends to decrease with distance from these “sampling hubs.” We reason that “sampling hubs” are highly accessible pentads, where highly active volunteers reside permanently (i.e., regular sampling in their home neighborhood) or temporarily (e.g., vacation accommodation when birding some distance from home), whereas the surrounding pentads require more effort to reach. To examine this, up to three sampling hubs were subjectively identified for each province (depending on the overall amount and pattern of sampling) and we calculated the distance between the midpoints of each pentad to the midpoint of the closest sampling hub (Figure 1, Table S1). The sampling hubs were not included in subsequent analyses.
2. Accessibility by road may facilitate long‐distance traveling to birding locations; therefore, the presence of roads is likely to increase sampling effort. We calculated the minimum distance between each pentad and a major road, that is, an arterial, national, main road, or freeway (Figure 1; AGIS, 2007). Lesser roads were not considered as they were found in almost all pentads.
3. Volunteers may associate protected areas with unspoilt natural scenic beauty and high biodiversity, in addition to infrastructure and facilities supplied by ecotourism, such as access roads and hiking trails, information, and accommodation (Tulloch et al., 2013). We calculated the percentage of each pentad covered by formal and informal protected area (Figure 1; SANBI 2010, 2011).
4. Volunteers may also be attracted to a range of bird habitats outside reserves, which may comprise various natural and human‐transformed land cover types. We focused here on percentage of a pentad covered by urban area and by cultivated area (the national land cover database, SANBI, 2009), as these are the two main transformed land cover types in all provinces and also tend to be collinear with natural land cover (i.e., negatively related).
5. Finally, volunteers may prefer certain climates and avoid, for example, extreme temperatures (e.g., Romo et al., 2006). We used mean annual precipitation, mean summer temperature, and mean winter temperature, averaged for each pentad, to examine preferred climatic conditions (Mecenero, Altwegg, Colville, & Beale, 2015; Schulze, 2001).

We used linear regression to determine how well variables 1–5 explain spatial variation in sampling effort represented by number of lists per pentad. “Distance to sampling hub” was log‐transformed to ensure a linear relationship with the response variable, because the untransformed relationship is a distance‐decay function. Separate generalized linear models for each province included all the explanatory variables listed, to examine their relative importance. Collinearity among predictors was generally low and never severe enough to justify excluding any predictors (O'Brien, 2007; see Variance Inflation Factors in Table S2). The models were fitted via penalized quasi‐likelihood using function “glmmPQL” in package “MASS” version 7.3‐45 (Venables & Ripley, 2002) in program R (R Core Team, 2016). We assumed a Poisson distribution and included an exponential spatial correlation structure as a random variable in each model to account for spatial autocorrelation.

### 2.3 Representativeness of data

**Figure 2:**
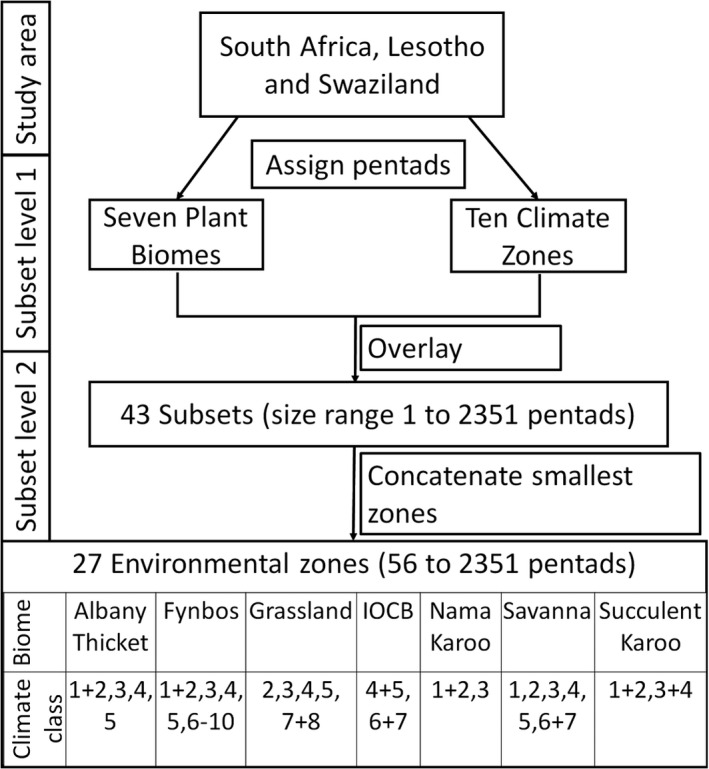
We defined 27 subsets of pentads to represent environmentally distinct zones. First, pentads were each assigned one of the seven biomes and one of the ten climate classes. The biome and climate classes were superimposed to form several climate zones within each biome, that is, altogether 43 environmental subsets comprised of varying numbers of pentads. Finally, the climate zones with the fewest pentads within each biome were pooled (symbol “+” indicates pooled subsets), resulting in a total of 27 environmental zones. IOCB refers to the Indian Ocean Coastal Belt, and climate classes range from hot and dry at “1” to moist and mild at “10.” See the main text and Figs. S1, S2a and S3 for more details on how climate classes were defined

We examined how variation in sampling intensity and the ability to detect the species that are present (sampling completeness) reflect in both geographical and environmental space. This idea is based on the potential for species distribution and occupancy models to estimate species distributions based on patchy species presence records and environmental background data (Bird et al., 2014; Bled et al., 2013; Guisan & Thuiller, 2005; Kramer‐Schadt et al., 2013). Bird distributions are driven by climate and vegetation type (Acevedo & Currie, 2003; Boone & Krohn, 2000; Van Rensburg, Koleff, Gaston, & Chown, 2004). Therefore, we defined environmentally distinct zones by partitioning all the pentads comprising South Africa, Lesotho, and Swaziland, into subsets of pentads with similar environments in terms of climate and vegetation biomes (for similar methods, see Robertson & Barker, 2006; Botts et al., 2011; Tulloch & Szabo, 2012). We first simplified the three climatic variables with a principal components analysis (PCA). The first PCA scores were related to mean annual precipitation, mean summer temperature, and mean winter temperature, with factor loadings 0.703, −0.675, and 0.225, respectively (Figs. S1 and S2a). The mapped component scores (Fig. S2a) show the main gradient between hotter, drier areas in the northwest, and milder, wetter areas in the southeast, as well as more local‐scale variations, such as at mountain ranges (see also Botts et al., 2011; Robertson and Barker, 2006). Therefore, although this component explains only about 57% of the variation, we deemed it a useful representation of climatic variation for our purposes. We then grouped the pentads into ten climate zones based on a histogram of the first PCA scores, which generally ranged from hot and dry at class 1 to moist and mild at class 10 (Figures 2, S1 and S2a).

Further, we assigned each pentad to the plant biome that covers the largest percentage of the pentad (Figures 2 and S2b). Mucina and Rutherford (2006) defined nine biomes, namely Desert, Nama Karoo, Succulent Karoo, Fynbos, Grassland, Savanna, Albany Thicket, the Indian Ocean Coastal Belt, and Forest (Fig. S2b). However, Forest and Desert comprised only a few pentads, and Mucina and Rutherford's (2006) Desert biome is mainly designated as Nama Karoo and Succulent Karoo in earlier vegetation maps for South Africa (e.g., Low & Rebelo, 1996; Rutherford, 1997; Rutherford & Westfall, 1994). Therefore, we assigned the Forest and Desert pentads to the closest neighboring biomes. Next, we superimposed the biomes and climate zones to define 43 distinct environmental zones of various geographical sizes (i.e., various numbers of pentads), representing several climate zones within each biome (Figure 2). That is, each biome was divided into several large (i.e., large number of pentads) climate zones that represent the typical climate range for that biome and several smaller zones that represent climate extremes for that biome.

Next, to quantify variation in sampling effort for each environmental zone, we counted all of the pentads with at least one list (i.e., total geographical coverage) as well as the pentads with ten lists or more (i.e., repeated samples necessary to model the observation process, Guillera‐Arroita et al., 2010). To examine sampling bias among environmental zones, we conducted *G*‐tests of independence comparing all pentads with sampled pentads, for both levels of sampling intensity, that is, at least one list and at least ten lists. To ensure that expected frequencies are above 5% in the *G*‐test, we pooled the smallest similar environmental zones within each biome to increase the number of pentads (Figure 2). This final process combining the smallest zones resulted in 27 environmental zones that were used for all further analyses (Figures 2 and S3).

We ranked the 27 zones according to sampling effort by calculating the difference between observed and expected frequency, where expected frequency is the number of lists that would have been contributed for each zone if sampling effort was geographically homogeneous. Expected frequencies were calculated for both levels of sampling intensity, using the following formula (see also Tulloch & Szabo, 2012): expected frequency = (number of pentads comprising an environmental zone ÷ total number of pentads) × total number of sampled pentads.

Assuming that number of species recorded would increase with number of pentads sampled (the species–area relationship), we used species accumulation curves to assess sampling completeness for each zone (see Moreno & Halffter, 2000; Tulloch & Szabo, 2012), for both levels of sampling intensity. For each zone, we calculated Mao Tau species richness estimates (R package “vegan,” version 2.4‐0, Oksanen et al., 2016), that is, a smoothed species accumulation curve produced by adding the pentads in random order (i.e., the average curve of 1,000 runs). We then fitted the Mao Tau estimates to an asymptotic Lomolino curve to estimate the total species richness (i.e., the asymptote) for each environmental zone (R package “vegan”; Lomolino, 2000; Dengler, 2009; Oksanen et al., 2016). We also tested the Clench and Weibull models (Hortal, Borges, & Gaspar, 2006; Moreno & Halffter, 2000; Tulloch & Szabo, 2012); however, the Lomolino model performed best in terms of fit and robustness. We then ranked the environmental zones according to sampling completeness by dividing each zone's observed species richness by the total estimated species richness, giving a percentage of completeness of the species inventory for that zone.

## 3 RESULTS

### 3.1 Determinants of spatial variation in sampling effort

The variables that best explained spatial variation in sampling effort varied somewhat among the provinces (Table 1, see Table S3 for more detailed results). Nevertheless, for most provinces sampling effort was significantly negatively related to distance to sampling hub (except for Limpopo and Northern Cape provinces) and distance to major road (except for Gauteng 4D and Mpumalanga Province), and significantly positively related to protected area cover (all provinces) and urban cover (except for Mpumalanga and North West provinces) (Table 1). Cultivated area, mean annual precipitation, mean summer temperature, and mean winter temperature were less important explanatory variables, being significant in only a few provinces (Table 1).

**Table 1:** Possible determinants of spatial variation in sampling effort in each province of South Africa. Distance to sampling hub and distance to major road represent accessibility, whereas the other variables describe the characteristics of grid cells. *T*‐values and level of significance are given

Province/Region	Distance to hub	Distance to road	Protected area	Urban area	Cultivation area	Mean annual precipitation	Mean summer temperature	Mean winter temperature
Gauteng 4D	−6.69****	−0.17 n.s.	4.64****	2.89**	0.66 n.s.	1.41 n.s	2.49*	0.13 n.s.
Mpumalanga	−4.34****	−1.83 n.s.	4.79****	1.43 n.s.	−1.06 n.s.	0.86 n.s.	1.86 n.s.	−0.91 n.s.
Limpopo	−1.66 n.s.	−4.12****	7.04****	2.31*	−2.75**	1.81 n.s.	2.23*	1.61 n.s.
North West	−9.73****	−2.72**	10.35****	0.71 n.s.	−0.58 n.s.	1.48 n.s.	1.65 n.s.	2.74**
Free State	−6.21****	−3.97***	6.73****	2.60**	−2.73**	1.69 n.s.	0.93 n.s.	0.73 n.s.
KwaZulu‐Natal	−4.22****	−4.49****	7.02****	4.01***	3.20**	1.81 n.s.	1.05 n.s.	−0.73 n.s.
Eastern Cape	−4.49****	−6.02****	5.42****	3.06**	1.88 n.s.	0.69 n.s.	1.16 n.s.	5.18****
Western Cape	−2.60**	−3.56***	3.07**	3.82***	1.15 n.s.	1.16 n.s.	1.08 n.s.	4.72****
Northern Cape	−0.61 n.s.	−5.23****	10.68****	3.70***	5.76****	3.22**	−0.13 n.s.	1.86 n.s.

Symbols: n.s. *p* > .05; *p < .05; **p < .01; ***p < .001; ****p < .0001.

### 3.2 Representativeness of data

**Figure 3:**
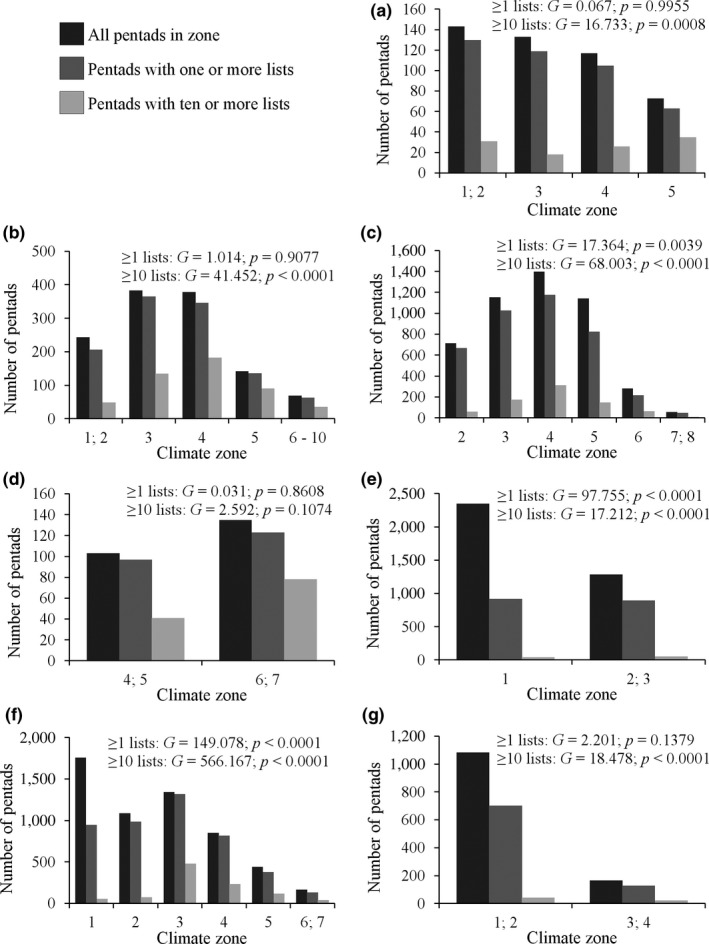
A comparison between the total number of pentads, the number of pentads that had been sampled at least once, and the number of pentads sampled at least ten times. This is shown for distinct environmental zones (subsets with varying numbers of pentads) comprising seven biomes, (a) Albany Thicket, (b) Fynbos, (c) Grassland, (d) Indian Ocean Coastal Belt, (e) Nama Karoo, (f) Savanna, and (g) Succulent Karoo, subdivided into ten climate zones, with the smallest zones concatenated. At each biome, we indicate the results of *G*‐tests of independence comparing all pentads with sampled pentads among the climate zones within each biome

The 27 environmental zones were not equally represented by pentads for both levels of sampling intensity (≥1 lists: *G* = 579.088, *p* < .0001; ≥10 lists: *G* = 1765.687, *p* < .0001; 26 degrees of freedom). For separate biomes, only the climate zones within the Albany Thicket, Fynbos, Indian Ocean Coastal Belt, and Succulent Karoo were evenly represented by pentads with at least one list, whereas only the Indian Ocean Coastal Belt's climate zones were evenly represented by pentads with ten lists or more (Figures 3 and 5; see also Fig. S4 for more details). The climate zones within the Grassland, Indian Ocean Coastal Belt, Fynbos, and Albany Thicket, and the wetter zones within the Savanna and Succulent Karoo have been especially well covered (more than 70% of pentads have been sampled at least once), with a substantial proportion of these pentads having ten or more lists (Figures 3 and 5, Table S4). However, the Nama Karoo's climate zones and the driest zones of the Succulent Karoo and Savanna are less well covered, and a negligible number of these pentads (fewer than 5% of pentads) have ten or more lists (Figures 3 and 5).

**Figure 4:**
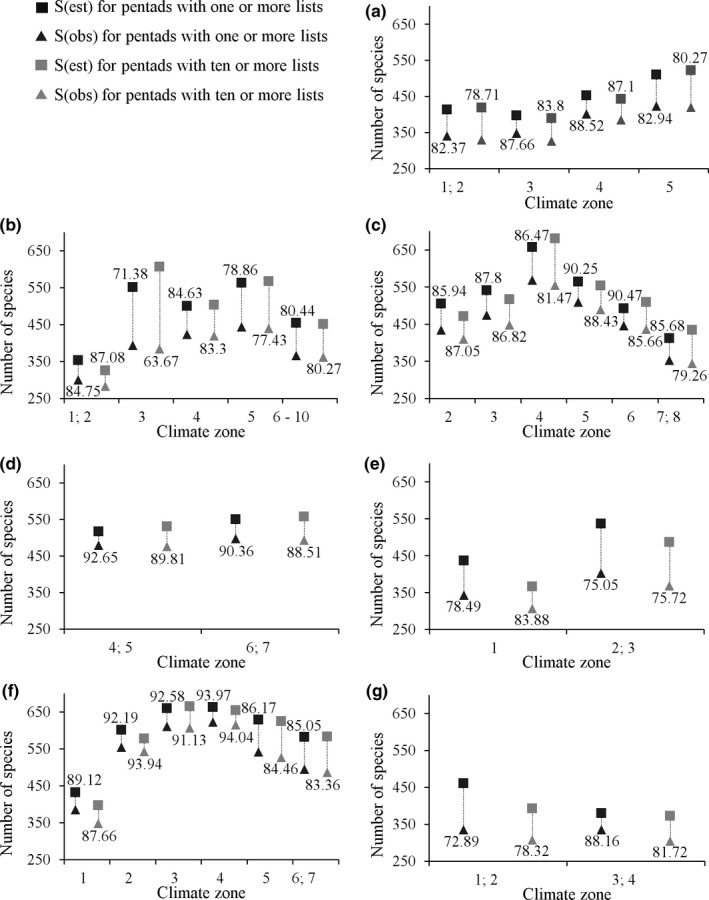
A comparison of observed, S(obs), and estimated, S(est), species richness for pentads that had been sampled at least once and at least ten times. This comparison was made for distinct environmental zones comprising seven biomes, (a) Albany Thicket, (b) Fynbos, (c) Grassland, (d) Indian Ocean Coastal Belt, (e) Nama Karoo, (f) Savanna, and (g) Succulent Karoo, subdivided into ten climate zones (Fig. S3). Labels indicate the ratio (as percentage) between S(obs) and S(est), which indicates the completeness of the species inventory for each environmental zone

**Figure 5:**
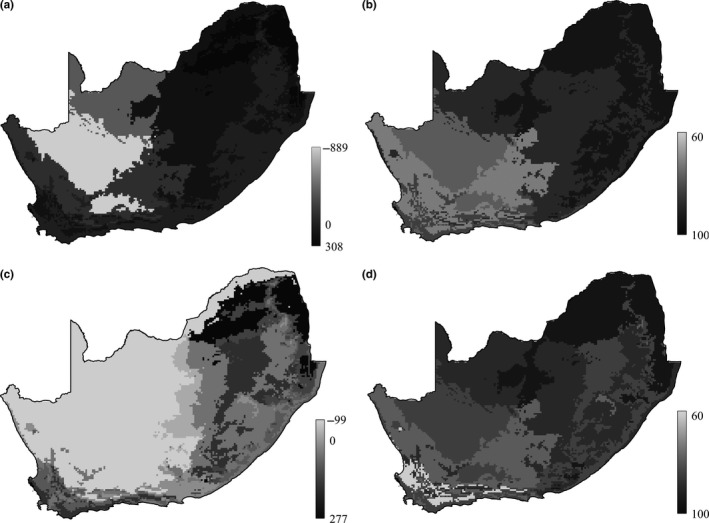
Environmental zones were ranked according to (1) sampling effort (a and c), that is, whether the zone had been sampled more or less than expected (number of lists contributed, see figure key) given the size of the zone and the overall number of lists contributed in the study area (Fig. S4) and (2) sampling completeness (b and d), that is, how much (percentage) of the total estimated number of species have been observed in each zone (Fig. S5). Two levels of sampling intensity were considered, namely, at least one list contributed per pentad (a and b) and at least ten lists per pentad (c and d)

The recorded species inventories for most of the environmental zones are more than 80% complete for both levels of sampling intensity, when comparing observed species richness to total estimated species richness given by the asymptote of the species accumulation curves (Figures 4 and S5, Table S4). Environmental zones were ranked differently (Figures 5, S4 and S5) when considering sampling completeness (i.e., species accumulation curves) compared to sampling effort (i.e., observed vs. expected sampling effort). For example, although the arid Savanna Zone is poorly sampled in terms of sampling effort, the species inventory is more than 87% complete because fewer species occur there (Figures 3, 4, 5). Conversely, climate zone 3 of the Fynbos biome is well sampled; however, its species inventory is <72% complete (Figures 3, 4, 5).

## 4 DISCUSSION

Volunteers are indispensable to the development of species atlases given the sheer magnitude of their contributed data and associated time, labor, and costs (Robertson et al., 2010; Tulloch et al., 2013). The second Southern African Bird Atlas Project covers an extensive geographical area, with large amounts of data especially for several subregions that are of special concern for bird diversity and conservation. However, like other species atlases (e.g., Botts et al., 2011; Tulloch & Szabo, 2012) SABAP2 is subject to pronounced spatial sampling bias, due to purposefully focused sampling in regions of special concern and due to the preferences of volunteers for certain sampling sites. Here we explored the causes and consequences of spatial variation in sampling effort, and we discuss current trends, strategies, and tools to mitigate bias and to improve the data accumulation process in species distribution atlases.

We found that variation in sampling effort is generally best explained by amount of urban area and protected area, and by the proximity of major roads, cities, and towns known for ecotourism (i.e., the “sampling hubs”). These findings agree with previous studies examining variation in sampling effort, including butterflies in the Iberian Peninsula (Romo et al., 2006), frogs in South Africa (Botts et al., 2011), and birds in Australia (Tulloch et al., 2013). In the current study, the importance of these determinants varied among the provinces (Table 1). For example, distance to nearest major road is not significant in the Gauteng 4D region, probably because of the relatively good road access (Figure 1, Tables 1 and S1). In contrast, distance to major road is important in the Northern Cape Province where the lack of major roads could restrict the movements of volunteers (Figure 1; Tables 1 and S1). Based on the overall results, we reason that many volunteers are likely resident in major cities and regularly conduct sampling in their own neighborhood and surroundings. When volunteers sample some distance from home, they prefer easy road access to a preferred destination such as a protected area or ecotourism town where they expect good birding opportunities. For the other southern African countries that we have not examined here, most areas remain unsampled and sampling appears to be closely linked to cities, towns, roads,. and other developed areas, or popular tourism destinations, more so than for South Africa (http://sabap2.adu.org.za/coverage.php, as viewed on 31 May 2017).

Spatial sampling bias affects how well the available data represent geographical and environmental space (Bird et al., 2014). We found that sampling coverage and intensity in current SABAP2 data are unequal among a set of distinct environmental zones across South Africa, Lesotho, and Swaziland. Large arid zones tend to be characterized by low sampling effort, unsampled gaps, and a small proportion of pentads with ten or more lists. Similar patterns were reported for other species distribution datasets in southern Africa (e.g., frogs, Botts et al., 2011; the first SABAP, Harrison & Underhill, 1997; plants, Robertson & Barker, 2006). The arid zones may be less attractive to volunteers due to expected low species richness and low accessibility to remote locations or private property (e.g., the mostly arid Northern Cape Province, Figure 1 and Tables 1 and S1; Tulloch et al., 2013). In contrast, wetter, milder environmental zones tend to coincide with the more densely populated areas of South Africa and have therefore been sampled more intensively, with a greater area covered and larger proportion of repeatedly sampled pentads. These well‐sampled zones also tend to be smaller compared to the arid zones, suggesting a higher turnover in environmental conditions across a smaller geographical area. Therefore, intensive sampling in these environmental zones may be beneficial and necessary to detect a higher species turnover and higher overall species richness that is often linked to environmental heterogeneity (Botts et al., 2011; Robertson & Barker, 2006; Van Rensburg et al., 2004). This is supported given that zones with high species richness and low species detectability may require a greater sampling effort (Figure 5; Garrard, Bekessy, Mccarthy, & Wintle, 2008; Wintle, Walshe, Parris, & Mccarthy, 2012).

Survey designs contend with a trade‐off between wider coverage of a geographical area and repeated sampling of representative sampling sites, depending on the objectives and the amount of sampling effort possible. SABAP2 currently comprises both wide‐coverage low‐intensity data and high‐intensity data from repeated sampling that are limited to certain geographical regions, biomes, and climates. Spatial and environmental sampling bias may have several consequences in terms of how well the spatial bias can be mitigated through data processing, how statistical and modeling techniques may be affected, and the type of ecological questions that can be adequately addressed (Bird et al., 2014; Guillera‐Arroita et al., 2015; Peterson et al., 1998). Therefore, researchers and conservation planners need to be aware of the region‐specific limitations of the data.

An environmental bias may affect the accuracy of species distribution and occupancy models that rely on environmental background data (Araújo & Guisan, 2006; Bird et al., 2014; Bled et al., 2013; Hernandez et al., 2006; Phillips et al., 2009). Wide‐coverage low‐intensity data are often used in broad‐scale species distribution modeling (Guillera‐Arroita et al., 2015). In addition, some species may not be present in the proportion of geographical and environmental space that had been repeatedly sampled, although they are likely to be observed through a wide‐coverage strategy covering a greater geographical area (Figure 4, Table S4). However, repeated sampling increases the probability of detecting the species that are present (Gu & Swihart, 2004). Moreover, sufficient repeated sampling is necessary to model the observation process (occupancy modeling), obtain abundance estimates, and examine species range dynamics (Bled et al., 2013; Broms et al., 2016; Guillera‐Arroita et al., 2015). Occupancy modeling can be refined with information about species' probability of detection (Guillera‐Arroita, 2017). Therefore, it would be useful to examine whether variation in detectability is predictable or quantifiable (Gu & Swihart, 2004), perhaps depending on environmental covariates (e.g., restricted visibility due to dense vegetation) or species traits (e.g., coexistence of species that are difficult to distinguish, or cryptic or nocturnal species).

Spatial biases in sampling effort may affect the conservation decision‐making process. For example, sampling bias in favor of regions with dense human populations may exaggerate any existing broad‐scale positive correlation between humans and bird species richness (Chown, Van Rensburg, Gaston, Rodrigues, & Van Jaarsveld, 2003; Evans et al., 2007; Van Rensburg et al., 2004). Consequently, conservation planning efforts often emphasize areas with high biodiversity near human settlements where there may be stronger competition between conservation goals and human development, while neglecting poorly sampled remote locations that may have high conservation potential (Evans et al., 2007). Further, an inability to account for the observation process could confound spatial changes in sampling effort with species range changes, in turn misrepresenting the species' conservation status (Broms, Johnson, Altwegg, & Conquest, 2014; Guillera‐Arroita et al., 2015; Péron & Altwegg, 2015).

The current study focused on natural environmental variation; however, future studies could examine sampling bias among land cover types. For example, relatively pristine and remote environmental zones might be underrepresented if data are mainly collected from the transformed areas within these zones, especially if species composition differs from the nearby natural environment (Dean, Anderson, Milton, & Anderson, 2002). For SABAP2, sampling effort in the arid zones tends to be close to human settlements and along roads (e.g., the Northern Cape Province, Table 1), that is, habitats that are atypical of the relatively untransformed arid zones. Over the past few decades, bird species such as pied crows (*Corvus albus*) that are native to more mesic areas of South Africa expanded their ranges into the arid areas, where they are associated with transformed areas and woody alien plants (Cunningham, Madden, Barnard, & Amar, 2016; Dean, 2000; Dean & Milton, 2003; Macdonald, 1986; Macdonald, Richardson, & Powrie, 1986). Increasing sampling in natural habitat may address this bias, and it may be helpful to incorporate land use as a covariate in species distribution models (Thuiller, Araújo, & Lavorel, 2004).

Recent developments in statistical methods provide many options for mitigating observation bias. However, ultimately, sampling bias should be actively monitored and addressed in all new or existing species distribution atlases. Wright et al. (2015) showed that SABAP2 volunteers are motivated by experiencing nature, recreation, personal growth, and the opportunity to contribute toward research and conservation. Communication and coordination among all participants are necessary to address sampling bias without sacrificing volunteer satisfaction and contribution (Bird et al., 2014; Sastre & Lobo, 2009). Atlas organizers play an essential role in maintaining volunteer participation by organizing a variety of birding events and challenges and supporting the online volunteer community (http://sabap2.adu.org.za). However, the link between volunteers and species atlases is becoming increasingly automatic and interactive. SABAP2 and other atlases such as eBird (http://ebird.org) link the online record submission process with tools to improve data accumulation (Kelling et al., 2013). Doubtful records, such as observing a bird species out of its known range, are automatically flagged during the submission process for vetting by experts (e.g., regional atlas committees). Online submissions are automatically linked to sampling effort coverage maps on the atlas Web sites, to inform volunteers' future sampling efforts. Species atlases and other citizen science projects benefit from increasingly sophisticated machine learning algorithms to facilitate the interaction between databases and volunteers (Kelling et al., 2013). Additional tools can be added to enhance the data accumulation process. For example, for an environmentally stratified sampling protocol, occupancy modeling could be applied to existing data to model the observation and detection processes and identify sampling sites to prioritize for additional sampling (Williams et al., 2009). Further, spatially predictable volunteer preferences could be taken into account when creating sampling coverage maps to encourage volunteers to visit priority sampling areas (current study, Tulloch et al., 2013). Thus, species atlasing is moving toward an iterative process whereby current data inform future priority sampling areas, and data accumulation is continually improved (Kelling et al., 2013).

## CONFLICT OF INTEREST

None declared.

## References

[ece33228-bib-0001] Acevedo, D. H. , & Currie, D. J. (2003). Does climate determine broad‐scale patterns of species richness? A test of the causal link by natural experiment. Global Ecology and Biogeography, 12, 461–473.

[ece33228-bib-0002] AGIS (2007). Agricultural Geo‐Referenced Information System. Retrieved from www.agis.agric.za

[ece33228-bib-0003] Altwegg, R. , Wheeler, M. , & Erni, B. (2008). Climate and the range dynamics of species with imperfect detection. Biology Letters, 4, 581–584. 18664423 10.1098/rsbl.2008.0051 PMC2610062

[ece33228-bib-0004] Araújo, M. B. , & Guisan, A. (2006). Five (or so) challenges for species distribution modelling. Journal of Biogeography, 33, 1677–1688.

[ece33228-bib-0005] Bird, T. J. , Bates, A. E. , Lefcheck, J. S. , Hill, N. A. , Thomson, R. J. , Edgar, G. J. , … Frusher, S. (2014). Statistical solutions for error and bias in global citizen science datasets. Biological Conservation, 173, 144–154.

[ece33228-bib-0006] Bled, F. , Nichols, J. D. , & Altwegg, R. (2013). Dynamic occupancy models for analyzing species' range dynamics across large geographic scales. Ecology and Evolution, 3, 4896–4909. 24455124 10.1002/ece3.858 PMC3892356

[ece33228-bib-0007] Boakes, E. H. , McGowan, P. J. K. , Fuller, R. A. , Chang‐qing, D. , Clark, N. E. , O'Connor, K. , & Mace, G. M. (2010). Distorted views of biodiversity: Spatial and temporal bias in species occurrence data. PLoS Biology, 8, e1000385. 20532234 10.1371/journal.pbio.1000385 PMC2879389

[ece33228-bib-0008] Boone, R. B. , & Krohn, W. B. (2000). Relationship between avian range limits and plant transition zones in Maine. Journal of Biogeography, 27, 471–482.

[ece33228-bib-0009] Botts, E. A. , Erasmus, B. F. N. , & Alexander, G. J. (2011). Geographic sampling bias in the South African Frog Atlas Project: Implications for conservation planning. Biodiversity Conservation, 20, 119–139.

[ece33228-bib-0010] Broms, K. M. , Hooten, M. B. , Johnson, D. S. , Altwegg, R. , & Conquest, L. L. (2016). Dynamic occupancy models for explicit colonization processes. Ecology, 97, 194–204. 27008788 10.1890/15-0416.1

[ece33228-bib-0011] Broms, K. M. , Johnson, D. S. , Altwegg, R. , & Conquest, L. L. (2014). Spatial occupancy models applied to atlas data show Southern Ground Hornbills strongly depend on protected areas. Ecological Applications, 24, 363–374. 24689147 10.1890/12-2151.1

[ece33228-bib-0012] Chown, S. L. , Van Rensburg, B. J. , Gaston, K. J. , Rodrigues, A. S. L. , & Van Jaarsveld, A. S. (2003). Energy, species richness, and human population size: Conservation implications at a national scale. Ecological Applications, 13, 1233–1241.

[ece33228-bib-0013] Cunningham, S. J. , Madden, C. F. , Barnard, P. , & Amar, A. (2016). Electric crows: Powerlines, climate change and the emergence of a native invader. Diversity and Distributions, 22, 1–13.

[ece33228-bib-0014] Dean, W. R. J. (2000). Alien birds in southern Africa: What factors determine success? South African Journal of Science, 96, 9–14.

[ece33228-bib-0015] Dean, W. R. J. , Anderson, M. D. , Milton, S. J. , & Anderson, T. A. (2002). Avian assemblages in native Acacia and alien *Prosopis* drainage line woodland in the Kalahari, South Africa. Journal of Arid Environments, 51, 1–19.

[ece33228-bib-0016] Dean, W. R. J. , & Milton, S. J. (2003). The importance of roads and road verges for raptors and crows in the Succulent and Nama‐Karoo, South Africa. Ostrich‐Journal of African Ornithology, 74, 181–186.

[ece33228-bib-0017] Dengler, J. (2009). Which function describes the species‐area relationship best? A review and empirical evaluation. Journal of Biogeography, 36, 728–744.

[ece33228-bib-0018] Evans, K. L. , Greenwood, J. J. D. , & Gaston, K. J. (2007). The positive correlation between avian species richness and human population density in Britain is not attributable to sampling bias. Global Ecology and Biogeography, 16, 300–304.

[ece33228-bib-0019] Garrard, G. E. , Bekessy, S. A. , Mccarthy, M. A. , & Wintle, B. A. (2008). When have we looked hard enough? A novel method for setting minimum survey effort protocols for flora surveys. Austral Ecology, 33, 986–998.

[ece33228-bib-0020] Gu, W. , & Swihart, R. K. (2004). Absent or undetected? Effects of non‐detection of species occurrence on wildlife–habitat models. Biological Conservation, 116, 195–203.

[ece33228-bib-0021] Guillera‐Arroita, G. (2017). Modelling of species distributions, range dynamics and communities under imperfect detection: Advances, challenges and opportunities. Ecography, 40, 281–295.

[ece33228-bib-0022] Guillera‐Arroita, G. , Monfort‐Lahoz, J. J. , Elith, J. , Gordon, A. , Kujala, H. , Lentini, P. E. , … Wintle, B. A. (2015). Is my species distribution model fit for purpose? Matching data and models to applications. Global Ecology and Biogeography, 24, 276–292.

[ece33228-bib-0023] Guillera‐Arroita, G. , Ridout, M. S. , & Morgan, B. J. T. (2010). Design of occupancy studies with imperfect detection. Methods in Ecology and Evolution, 1, 131–139.

[ece33228-bib-0024] Guisan, A. , & Thuiller, W. (2005). Predicting species distribution: Offering more than simple habitat models. Ecology Letters, 8, 993–1009. 10.1111/j.1461-0248.2005.00792.x 34517687

[ece33228-bib-0025] Guisan, A. , & Zimmermann, N. E. (2000). Predictive habitat distribution models in ecology. Ecological Modelling, 135, 147–186.

[ece33228-bib-0026] Harrison, J. A. , & Underhill, L. G. (1997). Introduction and methods In Harrison J. A., Allan D. G., Underhill L. G., Herremans M., Tree A. J., Parker V., & Brown C. J. (Eds.), The atlas of southern African Birds, Volume 1: Non‐passerines (pp. xliii–xliv). Johannesburg: BirdLife South Africa.

[ece33228-bib-0027] Harrison, J. A. , Underhill, L. G. , & Barnard, P. (2008). The seminal legacy of the Southern African Bird Atlas Project. South African Journal of Science, 104, 82–84.

[ece33228-bib-0028] Hernandez, P. A. , Graham, C. H. , Master, L. L. , & Albert, D. L. (2006). The effect of sample size and species characteristics on performance of different species distribution modeling methods. Ecography, 29, 773–785.

[ece33228-bib-0029] Hortal, J. , Borges, P. A. V. , & Gaspar, C. (2006). Evaluating the performance of species richness estimators: Sensitivity to sample grain size. Journal of Animal Ecology, 75, 274–287.16903065

[ece33228-bib-0030] Jetz, W. , McPherson, J. M. , & Guralnick, R. P. (2012). Integrating biodiversity distribution knowledge: Toward a global map of life. Trends in Ecology and Evolution, 27, 151–159. 22019413 10.1016/j.tree.2011.09.007

[ece33228-bib-0031] Kelling, S. , Lagoze, C. , Wong, W. K. , Yu, J. , Damoulas, T. , Gerbracht, J. , … Gomes, C. (2013). EBird: A human/computer learning network to improve biodiversity conservation and research. AI Magazine, 34, 10–20.

[ece33228-bib-0032] Kramer‐Schadt, S. , Niedballa, J. , Pilgrim, J. D. , Schröder, B. , Lindenborn, J. , Reinfelder, V. , … Wilting, A. (2013). The importance of correcting for sampling bias in MaxEnt species distribution models. Diversity and Distributions, 19, 1366–1379.

[ece33228-bib-0033] Lomolino, M. V. (2000). Ecology's most general, yet protean pattern: The species‐area relationship. Journal of Biogeography, 27, 17–26.

[ece33228-bib-0034] Low, A. B. , & Rebelo, A. G. (1996). Vegetation of South Africa, Lesotho and Swaziland. Department of Environmental Affairs and Tourism, Pretoria.

[ece33228-bib-0035] Macdonald, I. A. W. (1986). Range expansion in the pied barbet and the spread of alien tree species in southern Africa. Ostrich, 57, 75–94.

[ece33228-bib-0036] Macdonald, I. A. W. , Richardson, D. W. , & Powrie, F. J. (1986). Range expansion of the hadeda ibis *Bostrychia hagedash* in southern Africa. South African Journal of Zoology, 21, 331–342.

[ece33228-bib-0037] Mackenzie, D. I. , Nichols, J. D. , Royle, J. A. , Pollock, K. H. , Bailey, L. L. , & Hines, J. E. (2006). Occupancy estimation and modeling: Inferring patterns and dynamics of species occurrence. Amsterdam: Academic Press.

[ece33228-bib-0038] Mecenero, S. , Altwegg, R. , Colville, J. F. , & Beale, C. M. (2015). Roles of spatial scale and rarity on the relationship between butterfly species richness and human density in South Africa. PLoS One, 10, e0124327. 25915899 10.1371/journal.pone.0124327 PMC4411036

[ece33228-bib-0039] Moreno, C. E. , & Halffter, G. (2000). Assessing the completeness of bat biodiversity inventories using species accumulation curves. Journal of Applied Ecology, 37, 149–158.

[ece33228-bib-0040] Mucina, L. , & Rutherford, M. C. (2006). The vegetation of South Africa, Lesotho and Swaziland, Strelitzia 19. South African National Biodiversity Institute, Pretoria.

[ece33228-bib-0041] O'Brien, R. M. (2007). A caution regarding rules of thumb for variance inflation factors. Quality & Quantity, 41, 673–690.

[ece33228-bib-0042] Oksanen, J. , Blanchet, F. G. , Friendly, M. , Kindt, R. , Legendre, P. , McGlinn, D. , … Wagner, H. (2016). vegan: Community Ecology Package. R package version 2.4‐0. Retrieved from https://CRAN.R-project.org/package=vegan

[ece33228-bib-0043] Péron, G. , & Altwegg, R. (2015). Twenty‐five years of change in southern African passerine diversity: Nonclimatic factors of change. Global Change Biology, 21, 3347–3355. 25711802 10.1111/gcb.12909

[ece33228-bib-0044] Peterson, A. T. , Navarro‐Sigüenza, A. G. , & Benítez‐Díaz, H. (1998). The need for continued scientific collecting; a geographic analysis of Mexican bird specimens. Ibis, 140, 288–294.

[ece33228-bib-0045] Phillips, S. J. , Dudik, M. , Elith, J. , Graham, C. H. , Lehmann, A. , Leathwick, J. , & Ferrier, S. (2009). Sample selection bias and presence‐only distribution models: Implications for background and pseudo‐absence data. Ecological Applications, 19, 181–197. 19323182 10.1890/07-2153.1

[ece33228-bib-0046] R Core Team (2016). R: A language and environment for statistical computing. R Foundation for Statistical Computing, Vienna, Austria. Retrieved from https://www.R-project.org/

[ece33228-bib-0047] Reddy, S. , & Dávalos, L. M. (2003). Geographical sampling bias and its implications for conservation priorities in Africa. Journal of Biogeography, 30, 1719–1727.

[ece33228-bib-0048] Robertson, M. P. , & Barker, N. P. (2006). A technique for evaluating species richness maps generated from collections data. South African Journal of Science, 102, 77–84.

[ece33228-bib-0049] Robertson, M. P. , Cumming, G. S. , & Erasmus, B. F. N. (2010). Getting the most out of atlas data. Diversity and Distributions, 16, 363–375.

[ece33228-bib-0050] Romo, H. , García‐Barros, E. , & Lobo, J. M. (2006). Identifying recorder‐induced geographic bias in an Iberian butterfly. Ecography, 29, 873–885.

[ece33228-bib-0051] Rutherford, M. C. (1997). Categorization of biomes In Cowling R. M., Richardson D. M., & Pierce S. M. (Eds.), Vegetation of Southern Africa (pp. 91–98). Cambridge: Cambridge University Press.

[ece33228-bib-0052] Rutherford, M. C. , & Westfall, R. H. (1994). Biomes of southern Africa: an objective characterization. Memoirs of the Botanical Survey of South Africa, No. 63. National Botanical Institute, Cape Town.

[ece33228-bib-0053] SANBI (2009). National Land Cover (NLC). South African National Biodiversity Institute. Retrieved from http://bgis.sanbi.org/landcover/project.asp

[ece33228-bib-0054] SANBI (2010). NPAES Protected Areas (informal). South African National Biodiversity Institute. Retrieved from http://bgis.sanbi.org/protectedAreas/protectedAreas.asp

[ece33228-bib-0055] SANBI (2011). NBA 2011 Terrestrial Formal Protected Areas. South African National Biodiversity Institute. Retrieved from http://bgis.sanbi.org/NBA/terrestrial_formalprotecedareas.asp

[ece33228-bib-0056] Sastre, P. , & Lobo, J. M. (2009). Taxonomist survey biases and the unveiling of biodiversity patterns. Biological Conservation, 142, 462–467.

[ece33228-bib-0057] Schulze, R. E. (2001). South African atlas of agrohydrology and climatology. Water Research Commission, Pretoria, South Africa. Retrieved from http://planet.botany.uwc.ac.za/NISL/Invasives/Assignments/GARP/atlas/atlas.htm

[ece33228-bib-0058] Thuiller, W. , Araújo, M. B. , & Lavorel, S. (2004). Do we need land‐cover data to model species distributions in Europe? Journal of Biogeography, 31, 353–361.

[ece33228-bib-0059] Tulloch, A. I. T. , Mustin, K. , Possingham, H. P. , Szabo, J. K. , & Wilson, K. A. (2013). To boldly go where no volunteer has gone before: Predicting volunteer activity to prioritize surveys at the landscape scale. Diversity and Distributions, 19, 465–480.

[ece33228-bib-0060] Tulloch, A. I. T. , & Szabo, J. K. (2012). A behavioural ecology approach to understand volunteer surveying for citizen science datasets. Emu, 112, 313–325.

[ece33228-bib-0061] Van Rensburg, B. J. , Koleff, P. , Gaston, K. J. , & Chown, S. L. (2004). Spatial congruence of ecological transition at the regional scale in South Africa. Journal of Biogoegraphy, 31, 843–854.

[ece33228-bib-0062] Venables, W. N. , & Ripley, B. D. (2002). Modern applied statistics with S, 4th ed. New York: Springer.

[ece33228-bib-0063] Williams, J. N. , Seo, C. , Thorne, J. , Nelson, J. K. , Erwin, S. , O'Brien, J. M. , & Schwartz, M. W. (2009). Using species distribution models to predict new occurrences for rare plants. Diversity and Distributions, 15, 565–576.

[ece33228-bib-0064] Wintle, B. A. , Walshe, T. V. , Parris, K. M. , & Mccarthy, M. A. (2012). Designing occupancy surveys and interpreting non‐detection when observations are imperfect. Diversity and Distributions, 18, 417–424.

[ece33228-bib-0065] Wright, D. R. , Underhill, L. G. , Keenec, M. , & Knight, A. T. (2015). Understanding the motivations and satisfactions of volunteers to improve the effectiveness of citizen science programs. Society and Natural Resources, 28, 1013–1029.

