# Supplementary material for: The second Southern African Bird Atlas Project: Causes and consequences of geographical sampling bias
Source: Ecol Evol. 2017 Jul 27;7(17):6839–49. doi: 10.1002/ece3.3228 (PMC5587490; doi:10.1002/ece3.3228)

SUPPORTING FIGURES

Figure S1 We grouped pentads into ten climate zones ranging from hot and dry at ‘1’ to moist and mild at ‘10’. The classes were based on a histogram of the first component scores of a principal components analysis (PCA scores intervals indicated on the x axis). The first component was related to mean annual precipitation, mean summer temperature, and mean winter temperature, with factor loadings 0.703, -0.675, and 0.225, respectively. The minimum and maximum values for precipitation and temperature at each class is in the accompanying table.

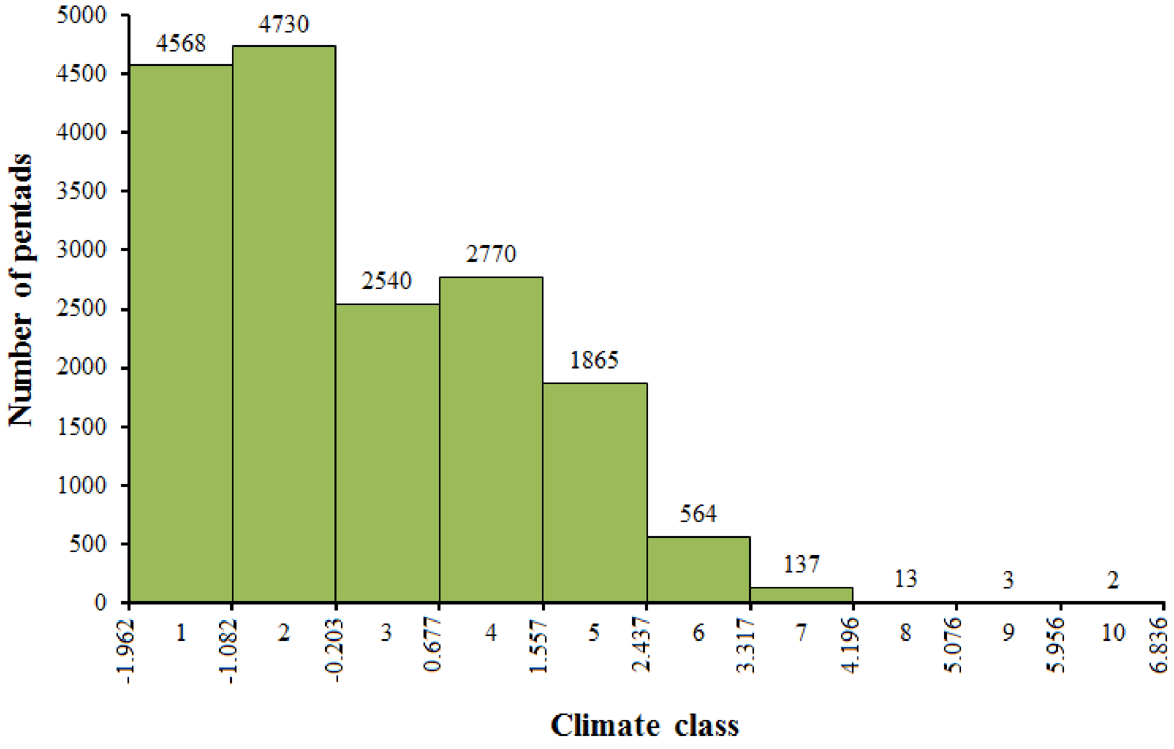

Climate class	Mean annual precipitation (mm)	Mean summer temperature (^{o}C)	Mean winter temperature (^{o}C)
1	22.28 - 453.00	29.76 - 34.00	-0.24 - 8.33
2	33.68 - 612.08	26.88 - 33.36	-1.00 - 10.00
3	37.80 - 759.28	24.76 - 32.52	-1.72 - 9.92
4	206.56 - 941.04	21.88 - 31.72	-1.16 - 12.00
5	451.32 - 1192.32	16.56 - 30.12	-6.64 - 13.00
6	492.20 - 1360.92	13.76 - 29.52	-8.16 - 12.76
7	527.40 - 1472.12	11.08 - 29.00	-9.04 - 13.00
8	919.68 - 1643.56	12.04 - 24.36	-9.64 - 6.00
9	1932.04 - 1931.04	21.48 - 21.48	3.32 - 3.32
10	2089.72 - 2310.12	21.52 - 22.16	4.96 - 5.20

Figure S2 Maps indicating variation environmental conditions across the study areas, in terms of (a) climate, based on the first component scores of a PCA (see Figure S1), and (b) plant biomes based on Mucina & Rutherford (2006). The colour shading in (a) is based on equal counts (quantile) to improve the clarity of the illustration.

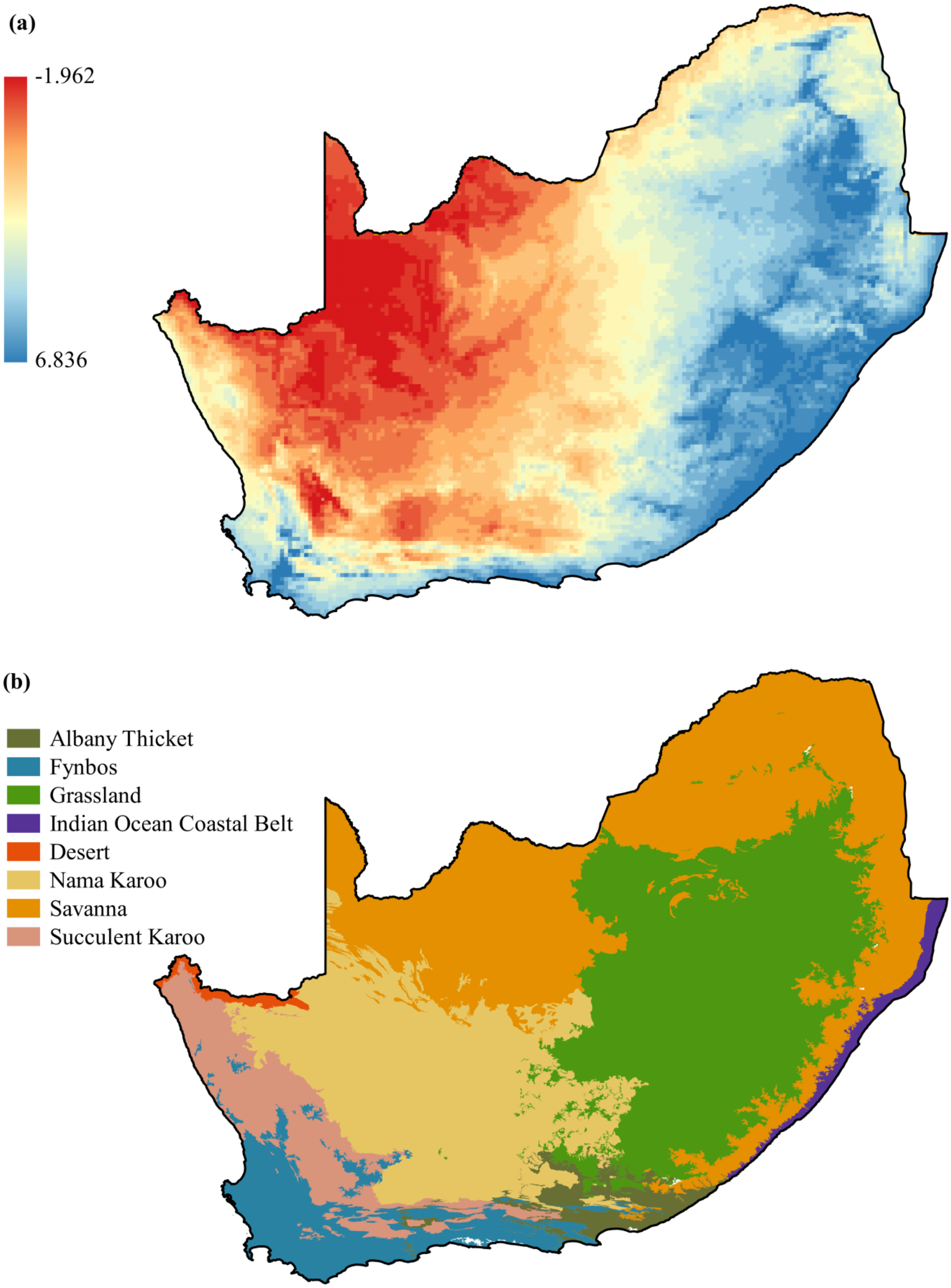

Figure S3 Pentads were grouped into environmental zones based on ten climate classes (labelled 1 to 10 in the key) superimposed on seven vegetation biomes (see Fig. S2). As indicated in the key, the smallest climate zones within each biome were pooled to ensure enough pentads in each zone for further analyses. See also Fig. 2 in the main text for a more detailed explanation of how environmental zones were defined.

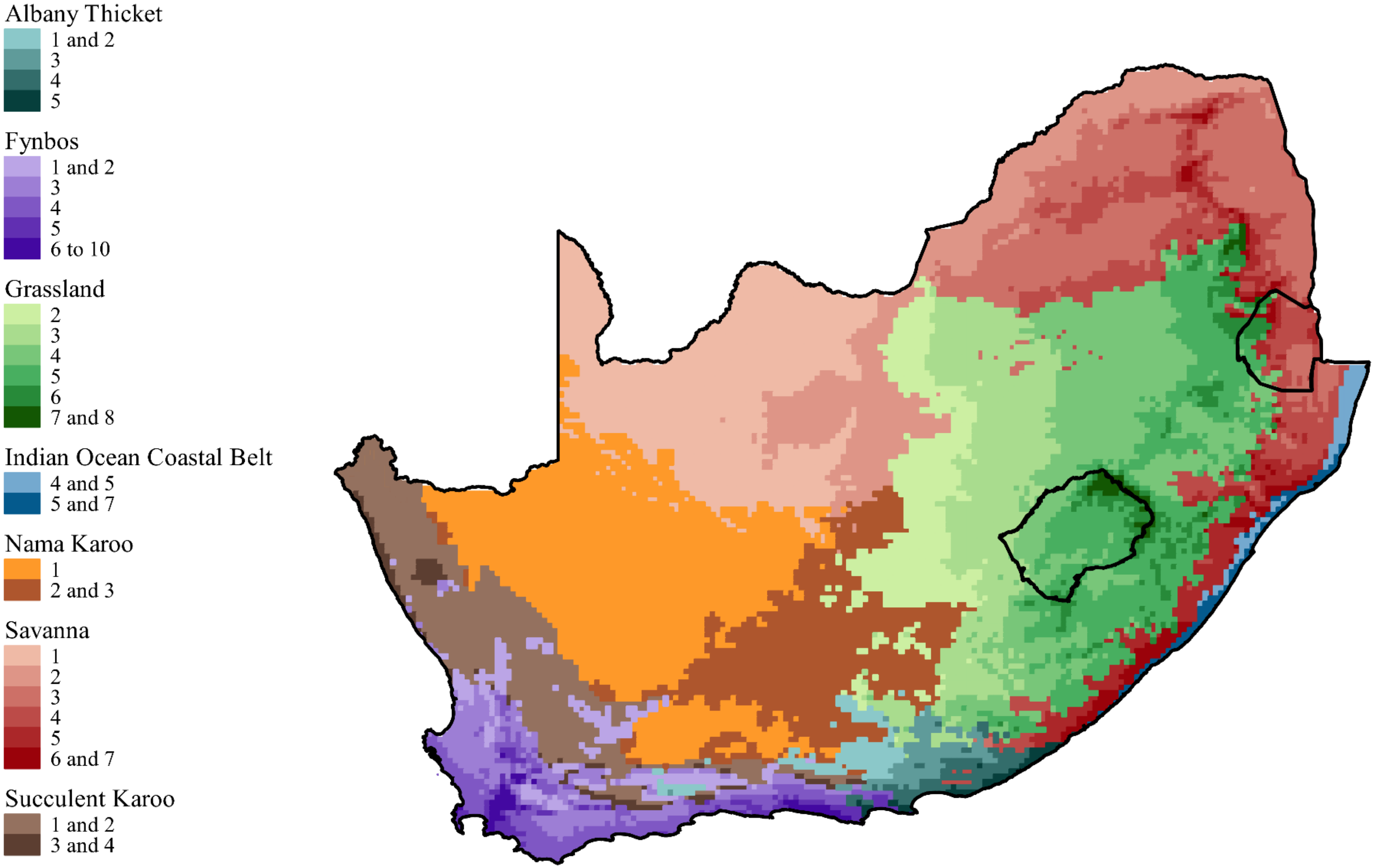

Figure S4 Variation in sampling effort (number of lists contributed) among distinct environmental zones, that is, whether the zone had been sampled more or less than expected. Expected frequency = (number of pentads for environmental zone ÷ total number of pentads) × total number of sampled pentads. Two levels of sampling effort were quantified, namely (a) one or more lists, and (b) ten or more lists. Note that the colour shading and Key is based on equal counts of pentads per class (quantile) to improve the clarity of the illustration.

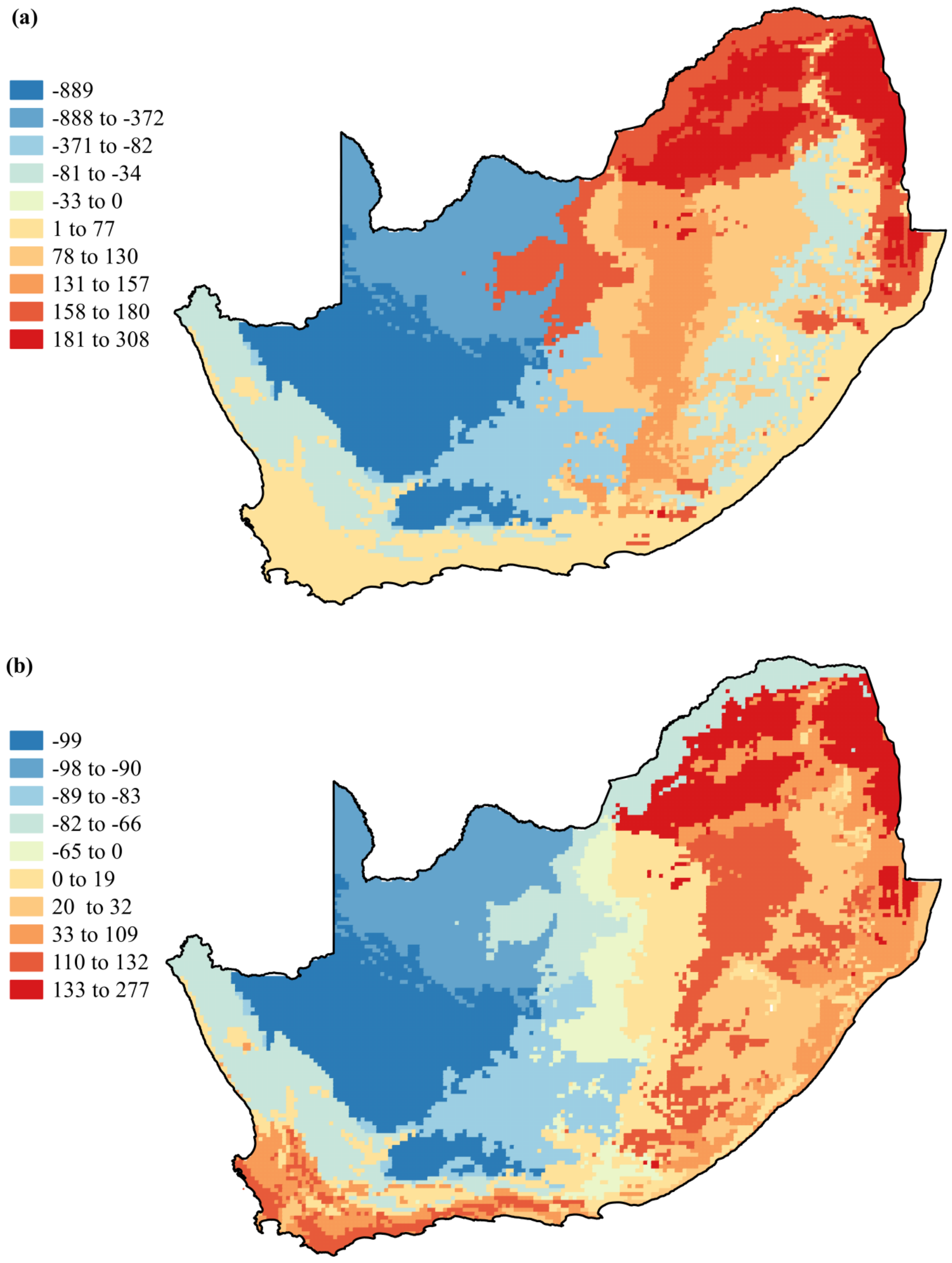

Figure S5 Variation in sampling completeness among distinct environmental zones, that is, how much (percentage) of the total estimated number of species have been observed in each zone. The percentage was based on the ratio between the observed species richness and the estimated total species richness given by the asymptote of species accumulation curves. Species accumulation curves were derived from pentads with (a) one or more lists, and pentads with (b) ten or more lists.

(a)

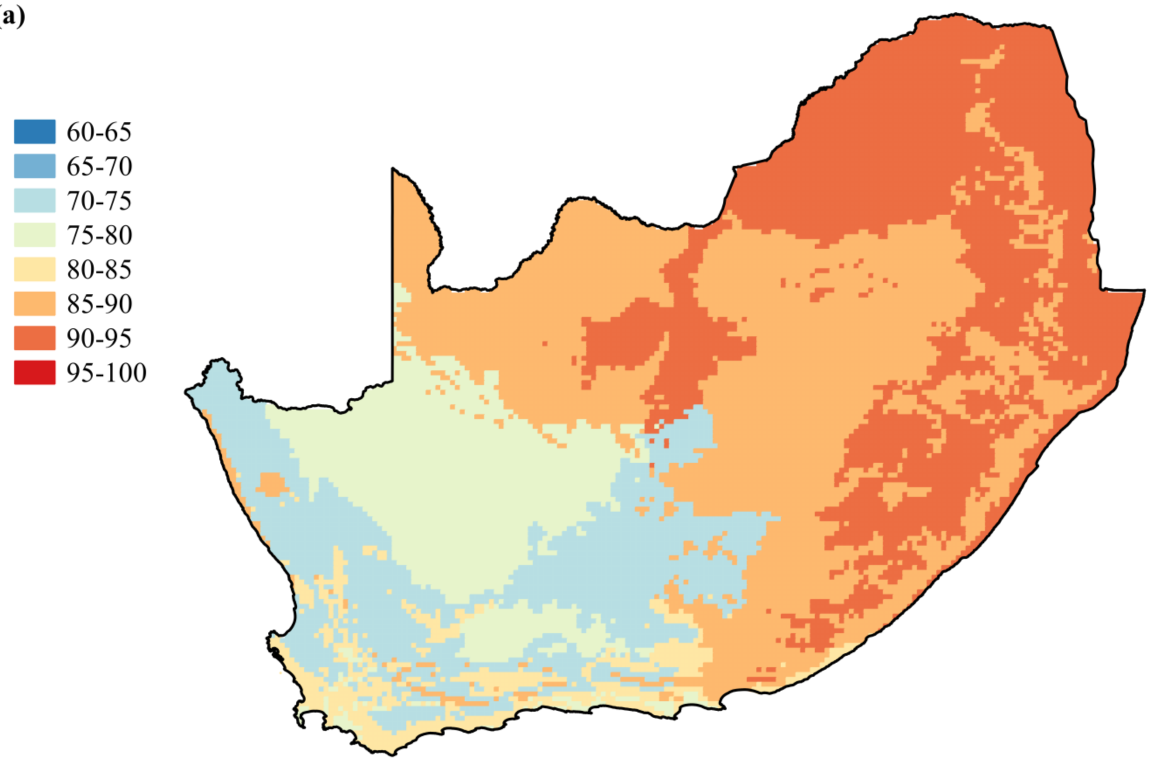

(b)

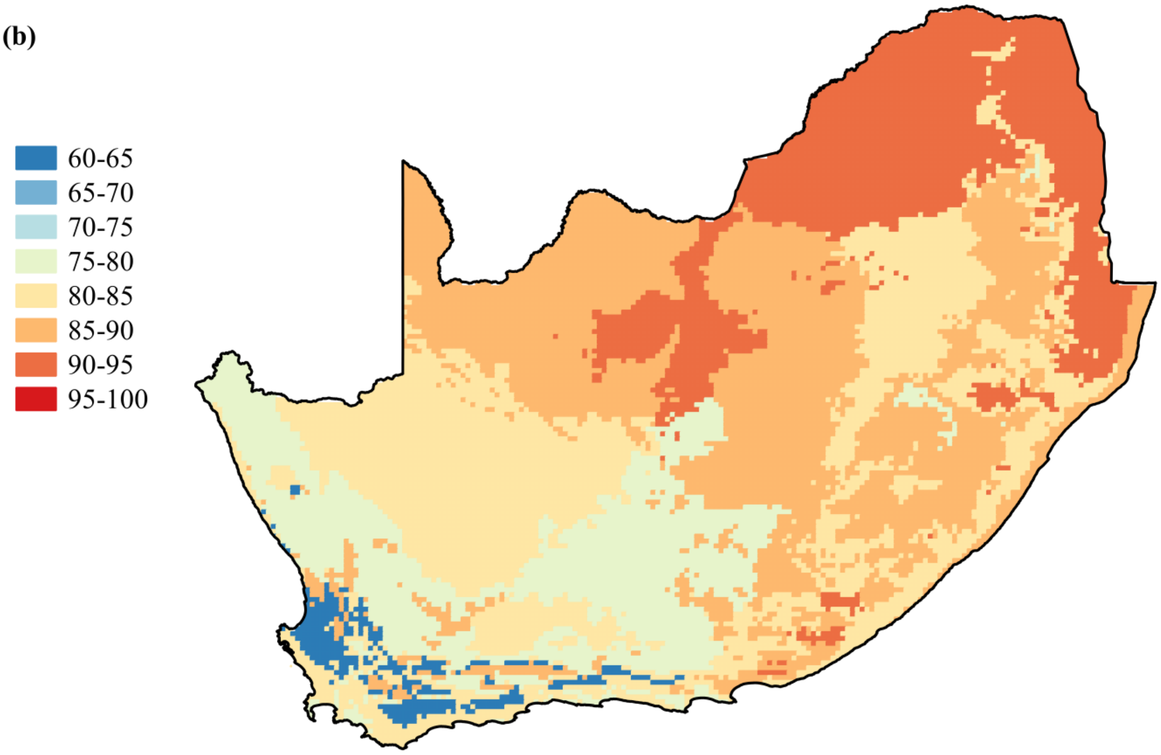

Supplement: Supplementary file 1 [file ECE3-7-6839-s001.pdf]
