# Supplementary material for: The second Southern African Bird Atlas Project: Causes and consequences of geographical sampling bias
Source: Ecol Evol. 2017 Jul 27;7(17):6839–49. doi: 10.1002/ece3.3228 (PMC5587490; doi:10.1002/ece3.3228)
Supplement: Supplementary file 2 [file ECE3-7-6839-s002.pdf]

Table S1 Details for each province: total number of lists (i.e. surveys), town or city closest to pentads with exceptionally high sampling effort ('sampling hubs'), proportion of pentads with access to major roads, mean and standard deviation of percentage cover per pentad of natural, urban, cultivated, and protected area, mean and standard deviation of annual precipitation, summer temperature and winter temperature.

Province	Total number of lists	Town or city closest to sampling hubs	Major Roads	Natural area (%)	Urban area (%)	Cultivated area (%)	Protected area	Mean annual precipitation	Mean summer temperature	Mean winter temperature
Gauteng4D	24167	Johannesburg, Pretoria (Rietvlei Nature Reserve)	0.686	56.73±23.07	7.91±13.49	28.78±20.84	5.32±16.27	639.7±5.32	27.93±1.54	1.10±1.09
Mpumalanga	10261	Middelburg, Nelspruit	0.485	64.53±25.83	2.11±5.79	17.30±19.92	17.46±34.80	736.44±169.72	26.52±2.63	3.72±2.98
Limpopo	11447	Modimolle	0.293	72.85±29.79	3.33±6.74	14.41±18.10	16.08±33.42	528.23±145.15	29.88±1.92	5.15±2.19
North West	4377	Klerksdorp	0.366	63.73±29.93	1.94±5.67	25.37±29.26	1.72±9.87	458.44±92.43	31.93±1.59	0.42±0.59
Free State	8052	Bloemfontein, Betlehem	0.457	63.69±26.65	0.73±3.91	32.26±26.65	1.70±9.23	532.98±125.79	28.64±2.13	0.03±0.45
KwaZulu-Natal	19007	Durban, Richards Bay	0.357	62.58±25.09	4.93±8.76	15.24±16.97	8.34±22.87	848.69±153.49	26.80±2.49	5.45±3.64
Eastern Cape	12471	Port Elizabeth, East London, Lady Grey	0.43	80.80±23.64	2.25±6.07	11.08±16.26	6.06±17.26	552.06±233.62	27.34±2.26	3.24±3
Western Cape	22970	Cape Town, Velddrif, Wilderness	0.398	78.71±29.77	1.42±7.75	17.31±27.07	13.88±26.36	348.35±227.13	28.82±2.18	4.15±2.14
Northern Cape	7269	Lime Acres, Port Nolloth	0.269	95.91±9.94	0.13±1.12	0.92±4.07	4.96±20.46	203.55±88.88	31.57±1.48	1.92±1.74
