# Supplementary material for: The second Southern African Bird Atlas Project: Causes and consequences of geographical sampling bias
Source: Ecol Evol. 2017 Jul 27;7(17):6839–49. doi: 10.1002/ece3.3228 (PMC5587490; doi:10.1002/ece3.3228)
Supplement: Supplementary file 3 [file ECE3-7-6839-s003.pdf]

Table S2 Variance Inflation Factors (VIF) for each predictor variable in each province, indicating collinearity between each predictor and all other predictors. A common rule of thumb about (VIF) states that values of 10 or more indicate serious collinearity, although this threshold is dependent on, e.g., sample size (O'Brien, 2007). Consequently, we decided that collinearity among predictors are acceptable and we included all predictors in the regression for each province.

	Distance to hub (log)	Distance to road	Protected area	Urban area	Cultivated area	Mean annual precipitation	Mean summer temperature	Mean winter temperature
Gauteng 4D	2.408	1.629	1.255	1.500	1.536	3.632	3.846	2.176
Mpumalanga	1.330	2.445	3.076	1.153	2.094	2.028	6.333	7.013
Limpopo	2.912	1.537	1.908	1.359	1.422	1.447	2.002	2.805
North West	4.449	1.300	1.053	1.034	1.441	6.001	8.511	1.857
Free State	1.123	1.174	1.037	1.066	1.236	5.518	5.746	1.171
KwaZulu-Natal	2.365	1.595	1.306	1.242	1.233	1.541	3.752	5.183
Eastern Cape	1.243	1.152	1.083	1.196	1.448	4.018	3.631	1.407
Western Cape	1.826	1.232	1.610	1.253	1.733	2.569	2.138	1.988
Northern Cape	1.451	1.251	1.217	1.067	1.115	2.742	1.069	2.761
