# Supplementary material for: The second Southern African Bird Atlas Project: Causes and consequences of geographical sampling bias
Source: Ecol Evol. 2017 Jul 27;7(17):6839–49. doi: 10.1002/ece3.3228 (PMC5587490; doi:10.1002/ece3.3228)
Supplement: Supplementary file 4 [file ECE3-7-6839-s004.pdf]

Table S3 Possible determinants of spatial variation in sampling effort in each province of South Africa. Degrees of freedom (*df*) is given for each regression (i.e. province). For each variable within each province, slope and standard error (SE), the t test statistic (*t*) and level of significance (*p*) are given.

	**Distance to hub**	**Distance to road**	**Protected area**	**Urban area**	**Cultivated area**	**Mean annual precipitation**	**Mean summer temperature**	**Mean winter temperature**
**Gauteng 4D (*df* = 564)**								
Slope ± SE	-1.078±0.161	-0.317±1.855	0.015±0.003	0.012±0.004	0.003±0.005	0.004±0.003	0.283±0.114	0.017±0.130
t	-6.689	-0.171	4.639	2.894	0.660	1.411	2.485	0.132
p	<0.0001	0.8643	<0.0001	0.0040	0.5098	0.1587	0.0133	0.8951
**Mpumalanga (*df* = 1035)**								
Slope ± SE	-0.684±0.158	-1.658±0.904	0.014±0.003	0.013±0.009	-0.006±0.006	0.001±0.001	0.175±0.094	-0.077±0.084
t	-4.341	-1.833	4.788	1.434	-1.060	0.855	1.857	-0.907
p	<0.0001	0.0670	<0.0001	0.1518	0.2894	0.3930	0.0636	0.3645
**Limpopo (*df* = 1650)**								
Slope ± SE	-0.384±0.232	-2.623±0.637	0.014±0.002	0.016±0.007	-0.013±0.005	0.001±0.001	0.151±0.068	0.103±0.064
t	-1.655	-4.117	7.035	2.309	-2.750	1.809	2.234	1.614
p	0.0982	<0.0001	<0.0001	0.0211	0.006	0.0706	0.0256	0.1068
**North West (*df* = 1421)**								
Slope ± SE	-1.266±0.130	-3.175±1.168	0.029±0.003	0.005±0.008	-0.002±0.003	0.004±0.002	0.230±0.140	0.560±0.204
t	-9.729	-2.718	10.347	0.713	-0.582	1.482	1.651	2.739
p	<0.0001	0.0067	<0.0001	0.4762	0.5609	0.1387	0.0989	0.0062
**Free State (*df* = 1799)**								
Slope ± SE	-0.780±0.126	-4.974±1.252	0.020±0.003	0.015±0.006	-0.008±0.003	0.002±0.001	0.079±0.085	0.163±0.223
t	-6.205	-3.973	6.727	2.601	-2.734	1.691	0.925	0.732
p	<0.0001	0.0001	<0.0001	0.0094	0.0063	0.091	0.3553	0.4646

**KwaZulu-Natal (*df* = 1369)**								
Slope ± SE	-0.572±0.136	-4.399±0.979	0.017±0.002	0.017±0.004	0.010±0.003	0.001±0.001	0.061±0.058	-0.032±0.044
t	-4.219	-4.494	7.016	4.011	3.197	1.805	1.054	-0.731
p	<0.0001	<0.0001	<0.0001	0.0001	0.0014	0.0713	0.2923	0.4651
**Eastern Cape (*df* = 2471)**								
Slope ± SE	-0.500±0.111	-8.620±1.431	0.014±0.003	0.013±0.004	0.008±0.004	0.0004±0.001	0.084±0.073	0.171±0.033
t	-4.493	-6.023	5.423	3.062	1.879	0.685	1.160	5.177
p	<0.0001	<0.0001	<0.0001	0.0022	0.0604	0.4936	0.2462	<0.0001
**Western Cape (*df* = 2005)**								
Slope ± SE	-0.337±0.130	-5.049±1.419	0.009±0.003	0.014±0.004	0.003±0.003	0.001±0.0004	0.059±0.054	0.329±0.070
t	-2.597	-3.558	3.070	3.819	1.149	1.161	1.080	4.715
p	0.0095	0.0004	0.0022	0.0001	0.2508	0.2459	0.2804	<0.0001
**Northern Cape (*df* = 5176)**								
Slope ± SE	-0.066±0.108	-2.545±0.487	0.020±0.002	0.051±0.014	0.025±0.004	0.003±0.001	-0.005±0.036	0.098±0.053
t	-0.610	-5.231	10.682	3.700	5.763	3.222	-0.134	1.858
p	0.5417	<0.0001	<0.0001	0.0002	<0.0001	0.0013	0.8933	0.0633
