# Supplementary material for: The second Southern African Bird Atlas Project: Causes and consequences of geographical sampling bias
Source: Ecol Evol. 2017 Jul 27;7(17):6839–49. doi: 10.1002/ece3.3228 (PMC5587490; doi:10.1002/ece3.3228)
Supplement: Supplementary file 5 [file ECE3-7-6839-s005.pdf]

Table S4 Sampling effort and sampling completeness for distinct environmental zones (climate zones 1 to 10 within biomes) and for two levels of sampling intensity. Zone size represents the number of pentads included in an environmental zone. Actual sampling effort represents the number of sampled pentads. Expected sampling effort indicates the number of sampled pentads expected given the size of the zone. Estimated species richness, S(est), was derived from the asymptote of a species accumulation curve, whereas S(obs) denotes the total number of species that had been observed in a zone.

**Biome**	**Climate**	**Zone size**	**At least one list per pentad (coverage)**				**Ten or more lists per pentad**			
			**Actual effort**	**Expected effort**	**S(est)**	**S(obs)**	**Actual effort**	**Expected effort**	**S(est)**	**S(obs)**
Albany Thicket	1,2	143	130	108	414	341	31	20	418	329
Albany Thicket	3	133	118	100	397	348	18	18	389	326
Albany Thicket	4	119	105	90	453	401	26	16	442	385
Albany Thicket	5	73	63	55	510	423	35	10	522	419
Fynbos	1,2	243	207	183	354	300	49	32	325	283
Fynbos	3	383	365	288	552	394	135	57	606	384
Fynbos	4	378	346	284	501	424	183	54	503	419
Fynbos	5	142	136	107	563	444	91	21	567	439
Fynbos	6 - 10	69	63	52	455	366	36	10	451	362
Grassland	2	715	668	538	505	434	61	104	471	410
Grassland	3	1155	1026	869	541	475	174	159	516	448
Grassland	4	1397	1175	1051	658	569	314	182	680	554
Grassland	5	1141	824	858	564	509	149	128	553	489
Grassland	6	282	218	212	493	446	66	34	509	436
Grassland	7,8	56	48	42	412	353	12	7	434	344
IOCB	4,5	103	97	77	517	479	41	15	530	476
IOCB	6,7	135	123	102	550	497	78	19	557	493
Nama Karoo	1	2351	918	1807	437	343	44	143	366	307
Nama Karoo	2,3	1284	893	975	537	403	56	139	486	368
Savanna	1	1758	950	1322	432	385	58	148	397	348
Savanna	2	1086	988	817	602	555	76	153	578	543
Savanna	3	1346	1320	1012	660	611	482	205	665	606
Savanna	4	850	819	639	663	623	236	127	654	615
Savanna	5	443	381	333	629	542	117	59	624	527
Savanna	6,7	169	135	127	582	495	40	21	583	486
Succulent Karoo	1,2	1084	703	765	461	336	43	109	392	307
Succulent Karoo	3,4	166	130	125	380	335	23	20	372	304
